# *Catharanthus roseus* Phytochemicals as Multi-Target Modulators of Disability-Linked Neurodegeneration: Bio-Computational Insights

**DOI:** 10.3390/ph18111734

**Published:** 2025-11-14

**Authors:** Qazi Mohammad Sajid Jamal, Ali H. Alharbi, Varish Ahmad, Khurshid Ahmad

**Affiliations:** 1 Department of Health Informatics, College of Applied Medical Sciences, Qassim University, Buraydah 51452, Saudi Arabia; 2 Health Information Technology Department, The Applied College, King Abdulaziz University, Jeddah 21589, Saudi Arabia; 3 King Salman Center for Disability Research, Riyadh 11614, Saudi Arabia

**Keywords:** *Catharanthus roseus*, molecular dynamics, disability, multi-target

## Abstract

**Background**: Disability-linked neurodegeneration involves cholinergic dysfunction, amyloidogenesis, glutamatergic excitotoxicity, and dopaminergic imbalance, highlighting the need for multi-target modulation. *Catharanthus roseus* contains a diverse array of metabolites with potential polypharmacological properties. **Methods**: We curated 318 *Catharanthus roseus* metabolites and performed structure-based virtual screening against five CNS targets, namely BACE1, AChE, MAO-B, NMDAR, and D1, using target-specific positive controls. Cross-target intersection ranking nominated three hits. We assessed dynamic stability by 200 ns all-atom molecular dynamics simulations (MDS) and MM/PBSA; ADMET-AI profiled CNS-relevant properties. **Results**: The three metabolites (PubChem CIDs 485711, 56964592, and 162963996) repeatedly ranked among top binders across targets. All five protein–ligand complexes reached stable MD plateaus (RMSD < ~0.30 nm) with sustained key interactions; BACE1 and AChE showed the highest contact persistence and most favorable ΔG_total/ligand-efficiency. **Conclusions**: Convergent docking, MDS, and MM/PBSA support these metabolites as tractable multi-target leads, with BACE1/AChE prioritized for enzyme-level validation and the remaining targets for follow-up studies.

## 1. Introduction

Neurological disorders are a significant contributor to disability worldwide, affecting individuals’ motor, sensory, cognitive, and functional abilities [1]. Despite progressions in medical interventions, many people continue to experience constant neurological difficulties that lead to substantial psychological and physical disabilities, limiting their quality of life and participation in society. Neurologically associated disability, such as Parkinson’s disease, Alzheimer’s disease (AD), and other cognitive losses, is due to defective cholinergic neurotransmission, caused by the lytic action of acetylcholinesterase (AChE) and butyrylcholinesterase (BChE) [2,3]. Therefore, therapeutic methods to manage the neurological disorder involve the modulation of the activity of BACE1 (Beta-site Amyloid Precursor Cleaving Enzyme 1), NMDAR (*N*-Methyl-D-aspartate Receptors), dopamine receptors, and inhibition of cholinesterase enzymes, AChE and BChE, and imparting better cholinergic functions of the brain. The increase in concentration of amyloid β plaques is a hallmark of AD, and BACE1 activity directly influences amyloid burden in the brain. NMDARs are glutamate-gated ion channels essential for learning, memory, and synaptic plasticity [4,5]. They facilitate the influx of calcium ions into neurons and accelerate signaling cascades that support long-term potentiation (LTP). An increase in NMDAR activity is associated with various neurological conditions, including neurodegeneration, while insufficient activity is reported to impair cognitive functions [6]. Dopamine receptors are G protein-coupled receptors, divided into D1-like (D1, D5) and D2-like (D2, D3, D4) families, which mediate dopaminergic signaling in the brain and regulate motivation, motor control, and cognition [7,8]. Targeting dopamine receptors remain a mainstay of pharmacotherapy in neurology and psychiatry. AChE is a catalytic protein that hydrolyzes acetylcholine in the synaptic cleft and influences the vital attention, memory, and learning processes. In AD, AChE inhibitors are used to enhance cholinergic function by inhibiting AChE-mediated breakdown of acetylcholine, thereby alleviating cognitive symptoms [9].

Recent research has explored the pharmaceutical potential of many phytochemicals. Alkaloids and phytochemicals of the alkaloid class of many plants indicate an auspicious frontier in the expansion of natural cholinesterase inhibitors for neurological-associated disorders. The Blending of traditional phytochemicals with contemporary pharmaceuticals may pave the way for more comprehensive and efficient methods of treating neurodegenerative illnesses and their impairments. The alkaloid profile of *Catharanthus roseus* (*C. roseus*) has been well explored, which includes vinblastine, vincristine, ajmalicine, and other indole alkaloids [10]. Traditionally, this plant has been recognized for its anticancer properties; recent studies indicate that certain phytochemicals from Catharanthus also exhibit neuroprotective activities [11]. One of the most extensively researched natural cholinesterase inhibitors is galantamine, an alkaloid derived from plants such as *Narcissus species* and *Galanthus nivalis* [12]. Huperzine A, derived from *Huperzia serrata*, and Berberine, an isoquinoline alkaloid found in plants such as Berberis species, have demonstrated AChE inhibitory activity, alongside anti-inflammatory and antioxidant effects [13]. The mechanisms of some alkaloids involve reversible binding to the active site or allosteric modulation, leading to increased acetylcholine levels in synaptic clefts [14]. Additionally, many of these compounds possess antioxidant, anti-inflammatory, and anti-amyloidogenic properties, providing multifaceted neuroprotection, particularly those derived *C. roseus* (Madagascar periwinkle), also known as Vinca, as natural cholinesterase inhibitors (CEIs). These plant-derived compounds, including alkaloids like vincristine and vinblastine, exhibit promising pharmacological activities, including neuroprotective effects mediated through cholinesterase inhibition [15,16].

In the evolving landscape of drug discovery, recent advances have underscored the pivotal role of structure-activity relationship (SAR) studies in refining the selectivity and potency of natural inhibitors. Furthermore, a comprehensive understanding of their pharmacokinetics and toxicity profiles remains essential to bridge the gap to effective clinical translation, paving the way for innovative therapies in chronic disease management [17]. Apart from alkaloids, flavonoids like quercetin and curcumin have shown inhibitory effects on AChE [18,19]. Recent investigations reveal that these phytochemicals can modulate cholinergic transmission and exhibit antioxidant activity, thereby providing neuroprotective effects [17,20]. The investigation of plant-derived alkaloids, particularly phytochemicals, as potential inhibitors of the cholinesterase enzyme has garnered considerable interest in recent neurological research. Exploring phytochemicals as cholinesterase inhibitors offers promising avenues for developing more effective, less toxic therapeutic agents for neurological disorders.

BACE1 contributes to amyloidogenic processing linked with synaptic failure and functional decline (disability endpoints), driving interest in disease-modifying approaches (e.g., human genetics/biomarker-linked trials) [21]. AChE targets cholinergic deficits; symptomatic AChE inhibition consistently improves cognition and daily activities, serving as a functional benchmark [22]. MAO-B regulates dopamine metabolism and glial ROS; clinically used MAO-B inhibitors support motor function in Parkinsonian disability and may affect non-motor areas [23,24]. NMDAR overactivation causes excitotoxic injury; clinically used uncompetitive antagonism (e.g., memantine-like) shows that controlled NMDAR modulation can preserve function [25]. D1 receptors influence frontostriatal circuits involved in working memory, motivation, and initiation; D1-targeted ligands impact executive and motor outcomes in preclinical and emerging clinical settings [26]. Taken together, these pathways directly correspond to cognitive and motor disability phenotypes, justifying their combined evaluation in a multi-target screen. We screened *318 C. roseus* metabolites across five druggable CNS targets, selected cross-target intersection hits for dynamic and energetic validation, and moved forward with in silico ADMET profiling.

## 2. Results and Discussion

Neurodegeneration underlying disability arises from convergent mechanisms, including cholinergic dysfunction, amyloidogenesis, glutamatergic excitotoxicity, and dopaminergic imbalance, suggesting that multi-target modulation may be advantageous over single-target strategies [27,28]. *C. roseus* is a rich source of neuroactive phytochemicals with potential polypharmacology [11]. We screened 318 *C. roseus* metabolites across five CNS targets under consistent conditions with target-specific positive controls, then prioritized the cross-target intersection (CIDs 485711, 56964592, 162963996) for dynamic and energetic validation. The IUPAC names and 2D structures of these hits are provided in Supplementary Table S1.

### 2.1. Targeting MAO-B

The MAO-B enzyme plays a crucial role in the catabolism of monoamine neurotransmitters, including dopamine, in the brain [29]. Increased MAO-B activity has been linked with elevated oxidative stress and neurodegeneration, contributing to the pathogenesis of neurological disorders like Parkinson’s disease. Inhibitors targeting MAO-B are used to raise dopamine levels and improve symptoms, highlighting the enzyme’s significance in therapeutic strategies for neurological disorders [30,31].

**Figure 1:**
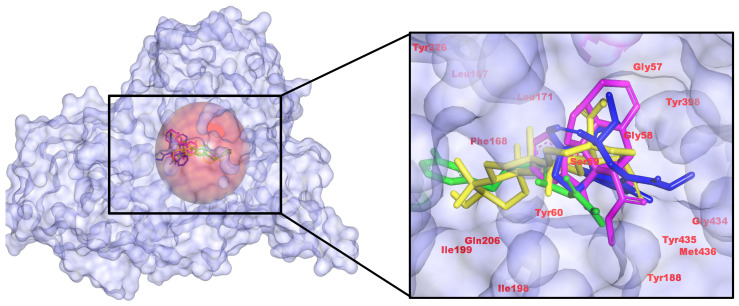
3D representation of *C. roseus* metabolites binding with MAO-B, highlighting critical hydrogen bonds to active site residues and pi interactions with aromatic amino acids. (**Left**): pocket surface with key residues labeled (catalytic/gating/anchor). (**Right**): overlay of the positive control (yellow) and the three *C. roseus* hits (consistent colors) with annotated H-bond/π distances. Colors: control (yellow), CID 485711 (green), CID 56964592 (magenta), CID 162963996 (blue).

All three *C. roseus* hits occupy the canonical substrate cavity and recapitulate the contact pattern of the positive control safinamide (CID 131682). Binding energies (kcal/mol) were −9.50 (control), −9.80 (485711), −10.02 (56964592), and −10.56 (162963996), indicating a modest but meaningful improvement for 162963996. Interaction maps show the control anchored by two H-bonds to Tyr60 with Tyr-rich π-packing; 485711 lacks classical H-bonds but exhibits strong aromatic/hydrophobic packing; 56964592 donates an H-bond to Gly434; 162963996 forms an H-bond to Met436. Taken together, pose quality and scoring nominate 162963996 as the leading MAO-B candidate, while acknowledging that docking energies are comparative rather than absolute (Figure 1). These features align with the established MAO-B pharmacophore, i.e., stacking within the Tyr398/Tyr435 aromatic cage and accommodation by the Ile199/Tyr326 gate, as defined in crystal structures (including the safinamide complex, PDB 2V5Z [32]) and multiple reviews [33]. Relative to safinamide, 162963996 preserves aromatic-cage engagement while shifting the H-bond anchor toward the backbone region (Gly434/Met436), an alternative noncovalent anchoring pattern reported for potent MAO-B inhibitors. This literature-consistent binding mode, together with its best docking score and favorable contact map, supports 162963996 as the leading MAO-B candidate [34].

### 2.2. Targeting AChE

Similarly, the identified three *C. roseus* hits bind within the AChE aromatic gorge and largely recapitulate the contact pattern of the positive control, donepezil (CID 3152), across active-site residues (Tyr72, Tyr124, Trp286, Tyr341, and Trp86) and the catalytic region (Ser203 and His447). Consistent with these poses, binding energies (kcal/mol) were −8.93 (control), −8.34 (485711), −10.49 (56964592), and −10.21 (162963996), indicating that 56964592 and 162963996 are the stronger AChE candidates.

**Figure 2:**
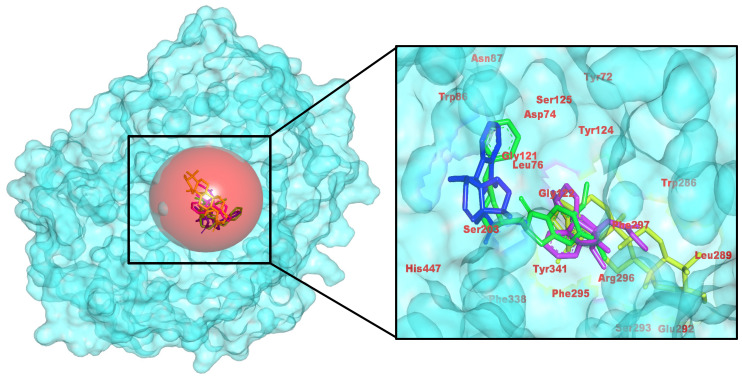
Atomic-level docked view in AChE, emphasizing hydrogen-bonding and π-π stacking by *C. roseus* natural products in the enzyme’s gorge. (**Left**): pocket surface with key residues labeled (catalytic/gating/anchor). (**Right**): overlay of the positive control (yellow) and the three *C. roseus* hits (consistent colors) with annotated H-bond/π distances. Colors: control (yellow), CID 485711 (green), CID 56964592 (magenta), CID 162963996 (blue).

Analysis of the docked complexes reveals that 56964592 (catharoseumine) establishes hydrogen bonds with Asp74 and Gly121/Tyr124. Compound 162963996 forms a hydrogen bond with Tyr72. Compound 485711 (epibetulinic acid) forms a single H-bond with Tyr341, accompanied by similar aromatic packing. In accordance with docking energies, 56964592 and, to a lesser degree, 162963996 demonstrate superior binding compared to the control, while 485711 exhibits moderate binding akin to the control (Figure 2). This pose reproduces the aromatic-gorge pharmacophore observed for human AChE–donepezil complexes—π–π/π-cation stacking to Trp86/Trp286 with Tyr-edge contacts (e.g., Tyr337/Tyr341) and a rim H-bond, matching canonical binding. Compared with bulky aryl-piperidines, our top hit retains the stacking core with a leaner H-bond pattern, consistent with efficiency-oriented SAR [35].

### 2.3. Targeting NMDAR

In line with the AChE and MAO-B results, all three *C. roseus* hits bind coherently within the NMDAR pocket and reproduce the control’s contact framework around active site residues. Binding affinities (kcal/mol) were −6.25 (control, CID 6604117), −6.58 (485711), −7.39 (56964592), and −6.95 (162963996), nominating 56964592 as the strongest NMDAR binder, with 162963996 also outperforming the control.

**Figure 3:**
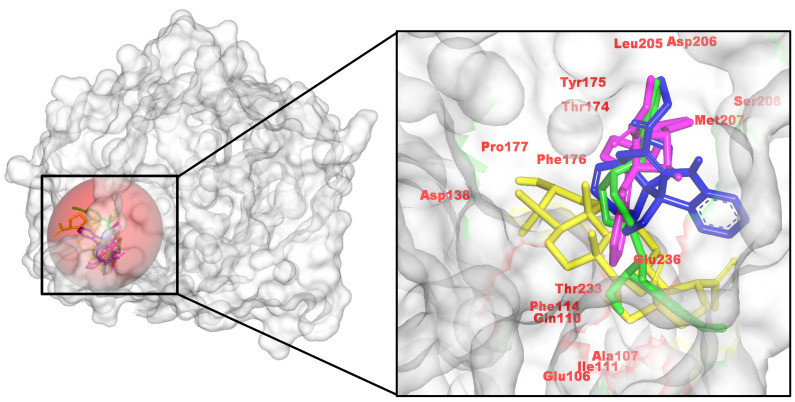
Spatial arrangements of *C. roseus* metabolites with the NMDAR, indicating a complex network of hydrogen and pi interactions for site anchoring. (**Left**): pocket surface with key residues labeled (catalytic/gating/anchor). (**Right**): overlay of the positive control (yellow) and the three *C. roseus* hits (consistent colors) with annotated H-bond/π distances. Colors: control (yellow), CID 485711 (green), CID 56964592 (magenta), CID 162963996 (blue).

Consistent with the AChE and MAO-B trends, all three *C. roseus* hits engage the NMDAR ligand-binding cleft spanning Glu106/Gln110 and the Thr174–Ser208 segment. 56964592 forms a multidentate H-bond network, with auxiliary polar contacts and π-alkyl packing. 162963996 makes H-bonds to Phe176 and Met207, plus a strong interaction with Glu236 and contacts to Asp206. 485711 donates H-bonds to Gln110 and Asp138 and packs against Ala107, Ile111, Phe114, and Pro178. In line with AutoDock Vina scores, 56964592 is prioritized as the top NMDAR binder, with 162963996 a close second (Figure 3). This binding pattern mirrors the cationic/H-bond anchor with hydrophobic enclosure described for NMDAR open-channel blockers, including memantine, as seen in cryo-EM structures of GluN1/2B (e.g., 7SAD) and related analyses of therapeutic channel blockers [36].

### 2.4. Targeting Dopamine D1 Receptor

In line with AChE, MAO-B, and NMDAR, all three *C. roseus* hits engage the D1 binding pocket and outperform the control. AutoDock Vina affinities (kcal/mol) were −6.4 (control, CID 6047), −8.3 (485711), −8.7 (56964592), and −7.5 (162963996), prioritizing 56964592 with 485711 closes behind.

**Figure 4:**
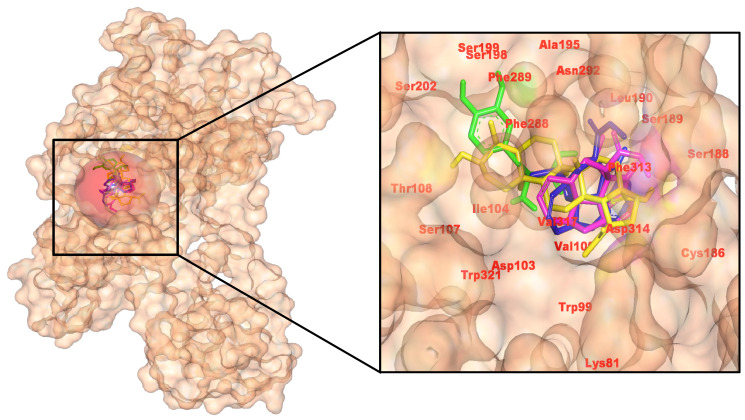
Docked arrangement of *C. roseus* compounds in Dopamine D1 receptor, detailing hydrogen bonds and π/alkyl interactions at ligand-binding regions. (**Left**): pocket surface with key residues labeled (catalytic/gating/anchor). (**Right**): overlay of the positive control (yellow) and the three *C. roseus* hits (consistent colors) with annotated H-bond/π distances. Colors: control (yellow), CID 485711 (green), CID 56964592 (magenta), CID 162963996 (blue).

Interaction analysis of 2D and 3D poses reveals that the control forms a small H-bond network (Ser198, Asp103, Ala195) with π-stacking at Phe289. 56964592 (catharoseumine) establishes three H-bonds (Leu190, Asn292, Ser188) and extensive hydrophobic/π contacts, consistent with its strongest score. 485711 (epibetulinic acid) donates H-bonds to Ser188 and Ser107, yielding a close second. 162963996 forms an H-bond with Ser188, giving an intermediate score. Collectively, pose quality and energies support 56964592 as the leading D1 binder, with 485711 also strong, reinforcing the multi-target potential of these *C. roseus* metabolites (Figure 4). The pose retains the aminergic orthosteric triad, ionic anchoring, H-bonding, and aromatic contacts as resolved in active-state D1 structures and GPCR pharmacology. A subtly shifted π-stack versus reference scaffolds is consistent with efficiency gains while preserving recognition [37].

### 2.5. Targeting BACE1

**Figure 5:**
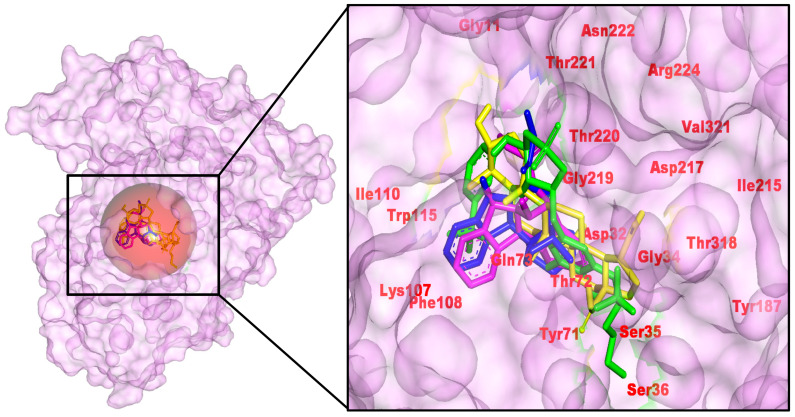
Docked poses of control and *C. roseus* hits in the BACE1 active site (PDB 4D8C). Left, surface view of BACE1 with the binding pocket circled; right, zoom-in showing overlaid poses within the catalytic cleft. Labeled residues mark the catalytic region, gating residues, and pocket-lining residues. (**Left**): pocket surface with key residues labeled (catalytic/gating/anchor). (**Right**): overlay of the positive control (yellow) and the three *C. roseus* hits (consistent colors) with annotated H-bond/π distances. Colors: control (yellow), CID 485711 (green), CID 56964592 (magenta), CID 162963996 (blue).

BACE1 has been known to cleave the amyloid precursor protein (APP) at the β-secretase site to initiate the construction of Aβ peptides. These accumulate to form toxic oligomers as well as the amyloid plaques, which are associated with AD [38]. In our overlay (Figure 5), the three *C. roseus* hits adopt conserved orientations within the catalytic cleft, aligning with the control inhibitor (CID 44625397) and filling the S1–S3 sub pockets, which are framed by the gating Tyr71/Thr72 loop. Across engines, the hits outperformed the control: Vina/AD4 energies were −9.64/−9.17 (56964592), −9.50/−8.82 (485711), and −9.20/−8.79 (162963996) versus −8.20/−7.93 for the control, translating to AD4-estimated Ki values of ~190 nM, 345 nM, and 359 nM, respectively (control 1.55 µM) (Table 1). While docking energies are comparative rather than absolute, the consistent ∼0.8–1.2 kcal·mol^−1^ gains and sub-micromolar Ki estimates nominate these metabolites as credible BACE1 binders.

Interaction analysis supports the scoring trends. The control forms a multi-point H-bond network with Thr72, Tyr187, Asp32, Gly34, Gly219, reinforced by halogen/π contacts. 56964592 (catharoseumine) preserves catalytic contacts and adds H-bonding to Gln73. 485711 (epibetulinic acid) forms H-bonds to and packs hydrophobically against Asp32, Tyr71, Ser35, Gly219, Thr220, Asp217/Arg224, with additional alkyl contacts to Ile110/Ile215/Val321. 162963996 H-bonds to Thr221, with π-lone-pair/π-alkyl stabilization from Gln73/Tyr71. The common engagement of Asp32 (catalytic), Tyr71/Thr72 (gating), and Thr220/221/Gly219 (pocket wall) rationalizes the observed ranking and indicates chemically plausible occupancy of the active site. To avoid over-interpreting single-frame docking metrics, we treat ΔE solely as a comparative metric and rely on MD-averaged persistence and MM-PBSA/LE trends for ranking. Using this multi-metric approach, 162963996 and 56964592 remain the most promising BACE1 chemotypes. The complex aligns with the Asp32/Asp228 catalytic dyad and stabilizes the flexible Tyr71 “flap”, a hallmark of potent BACE1 binders, as revealed by structural/mechanistic studies. Compared with diol-rich isosteres, the hit favors flap engagement with a moderated donor count, a balance often linked to improved efficiency while preserving catalytic-site geometry [39,40].

Given the concordance between pose quality and energetics, and BACE1’s causal role in Aβ biogenesis, we prioritized these three complexes (plus the control) for dynamic and thermodynamic validation. The subsequent 200 ns MD, essential dynamics/FEL, and MM-PBSA analyses, targeting the two most prominent proteins, AChE and BACE1, test whether the favorable docking poses translate into sustained active-site engagement and comparatively improved binding free energies.

**Figure 6:**
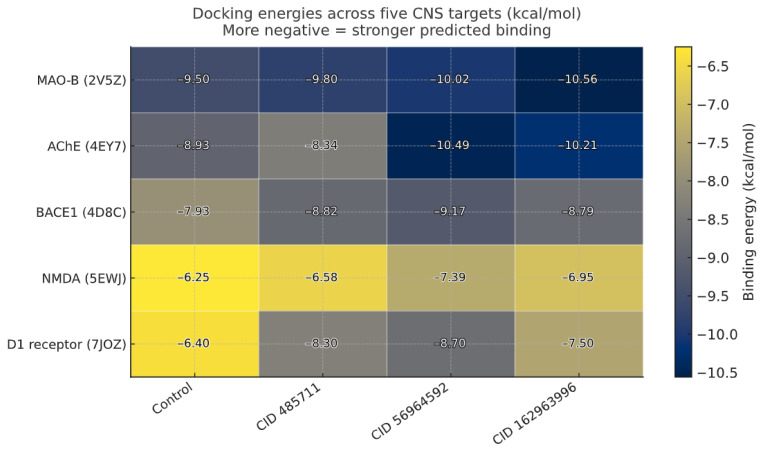
Consolidated docking heatmap: Docking energies for the target-specific control and *C. roseus* hits against selected targets. More negative values indicate stronger predicted binding; numeric energies are overlaid in each cell. Darker shading indicates more favorable binding.

Taken together, the docking and interaction analyses indicate a coherent polypharmacology signal, with the three *C. roseus* metabolites repeatedly ranking among the top ligands across BACE1, AChE, MAO-B, NMDAR, and D1 (Figure 6). Catharoseumine (CID 56964592) most consistently matches or surpasses each target’s positive control, while CIDs 162963996 and 485711 show target-selective strength. This pattern is mechanistically plausible—BACE1’s catalytic cleft and the AChE aromatic gorge favor the π-stacking/H-bond networks seen in our poses. In contrast, NMDAR and D1 rely more on complementary hydrogen bonds and hydrophobic packing within their ligand pockets. Because BACE1 is the initiating β-secretase for Aβ production and a prime disease-modifying target in early AD [41], we prioritized it for deeper validation with MD, essential-dynamics/FEL, and MM-PBSA.

Our findings align with prior reports that plant-derived polyphenols and alkaloids can modulate these same pathways. Multiple natural products reduce Aβ by lowering BACE1 activity or expression, including curcumin and EGCG (cell and animal studies), as well as resveratrol scaffolds that directly inhibit β-secretase activity. Ginsenosides (e.g., Rg1) also decrease Aβ levels via BACE1-related mechanisms. In the cholinergic axis, galantamine (and huperzine A) exemplify phytochemical AChE inhibitors used clinically or investigated in trials. On the excitotoxicity axis, memantine, a clinically approved NMDAR antagonist, demonstrates that moderate-affinity, uncompetitive blockade can be therapeutically effective. At the same time, the D1 receptor remains a promising target for cognitive and motor symptoms. These precedents support the plausibility that *C. roseus* metabolites can act as multi-target leads for AD and related neurodisabilities [9,42,43].

Given the inherent limitations of docking, including scoring-function bias and limited receptor flexibility, we next assessed whether the favorable poses translate to dynamic stability and favorable binding energetics in a solvated, flexible environment. Accordingly, we advanced the 200 ns MDS for all selected receptors, essential dynamics/FEL, and MM-PBSA analyses of these hits on BACE1 to test the persistence of key contacts, conformational stabilization, and relative free-energy trends.

### 2.6. MD Simulations

**Figure 7:**
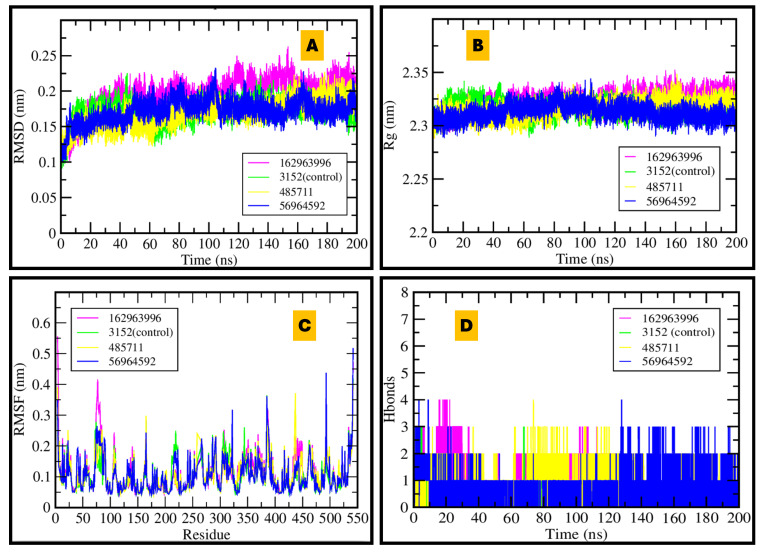
MDS of AChE–ligand complexes over 200 ns: (**A**) RMSD shows system equilibration; (**B**) stable radius of gyration; (**C**) residue-wise RMSF revealing flexibility hotspots; (**D**) number of hydrogen bonds, with 56964592 achieving the highest persistence.

**Figure 8:**
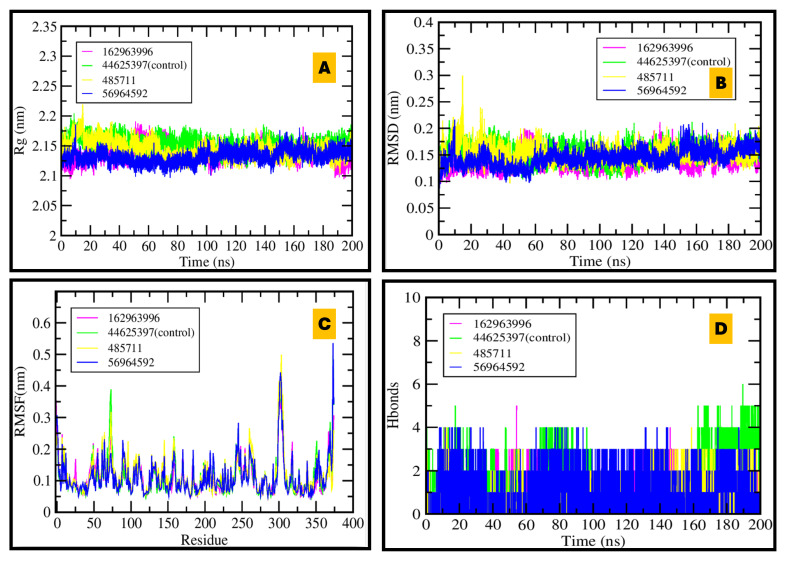
MDS of BACE1–ligand complexes over 200 ns: (**A**) Stable radius of gyration demonstrates compact protein structure; (**B**) RMSD plot shows good equilibration and dynamic stability; (**C**) RMSF indicates flexibility is restricted to specific regions; (**D**) Hydrogen bond plot reveals that 56964592 and control inhibitor maintain the most persistent protein–ligand interactions.

RMSD, RMSF, hydrogen bonding, and radius of gyration are key parameters used to conclude protein-ligand stability during molecular simulations. The observed results of both (AChE and BACE1) complexes are shown in Figure 7 and Figure 8. Stable treatments are more likely when MDS reveals whether robust docking to the BACE1 catalytic dyad (Asp32, Asp228) is maintained dynamically and confirms that strong docked positions are not a result of vacuum or rigid settings.

#### 2.6.1. AChE

AChE MDS plots showed stable RMSD (<0.25 nm) for all complexes over 200 ns, indicating minimal structural drift. The radius of gyration (Rg ≈ 2.3 nm) remained flat, consistent with retained compactness. RMSF revealed low residue flexibility with only loop-level fluctuations; notably, CID 162963996 exhibited a slight increase in local mobility without significant rearrangement. Hydrogen-bond timelines identified CID 56964592 (catharoseumine) as having the highest persistence, supporting a durable anchoring network. These findings align with the docking rank-bands and nominate 56964592 as the most robust AChE binder, with 162963996 also maintaining sustained contacts (see Figure 7 for RMSD/Rg/RMSF/H-bond panels; Supplementary Figures S1 and S3–S5 for water bridges, SASA, RDF, and occupancy timelines).

#### 2.6.2. BACE1

For BACE1, Rg remained ~2.1–2.3 nm over 200 ns, indicating preserved fold integrity. RMSD stabilized rapidly within 0.1–0.2 nm, and RMSF profiles were uniformly low apart from expected loop/termini fluctuations. Hydrogen-bond analysis highlighted CID 56964592 and the control inhibitor as the complexes with the most persistent interactions, consistent with sustained engagement near the Asp32/Asp228 catalytic dyad. These data support the docking-inferred ranking and identify 56964592 as a leading BACE1 antagonist candidate (see Figure 8 for RMSD/Rg/RMSF/H-bond panels; Supplementary Figures S1 and S3–S5 for hydration/occupancy metrics).

Beyond global RMSD/Rg/RMSF, interaction persistence and solvation were quantified using time-fraction contact occupancy, SASA, RDF, and water-bridge dynamics (Supplementary Figures S1 and S3–S5), demonstrating sustained active-site contacts for 56964592 and 162963996.

Consistent stability patterns were also observed for MAO-B, NMDAR, and D1 in 200 ns trajectories (Supplementary Figures S6–S8), where RMSD/Rg stayed within narrow ranges and hydrogen-bond timelines supported persistent binding, thereby strengthening the multi-target assertion with a clear main-text summary and detailed information in the Supplement. See Figures S6–S8 for MAO-B, NMDAR, and D1 MD panels; solvation and occupancy metrics in Figures S1 and S3–S5.

### 2.7. MMPBSA Analysis

End-state binding free energies (ΔG_bind_) were estimated with gmx_MMPBSA using both Generalized Born (GB) and Poisson–Boltzmann (PB) continuum models over the last 100 ns of each 200 ns trajectory (block-averaged, 5 × 20 ns blocks). For BACE1, the positive control yielded −20.85 ± 0.07 kcal·mol^−1^ (mean ± SEM), while the three *C. roseus* hits fell in the −14.64 to −16.09 kcal·mol^−1^ range (see Table 2 for GB values; PB components in Table S2). These magnitudes are consistent with the MD-observed pose persistence and the docking rank-bands reported earlier.

Because end-state MM-PB/GBSA is most reliable for relative comparisons, we treat ΔG_bind_ as a comparative metric rather than an absolute potency estimate. Where SEM-based uncertainty suggests overlap (i.e., differences comparable to the SEM; approximate 95% CIs ≈ mean ± 1.96 × SEM), compounds are considered statistically indistinguishable and are prioritized using additional, orthogonal evidence: (i) high-occupancy MD contacts during the 100–200 ns window, and (ii) ligand efficiency (LE = −ΔG_bind_/N_heavy_). We report GB-model ΔG_bind_ and SEM in Table 2 and provide PB-model components with SD alongside SEM in the Supplement (Table S2). LE values are summarized in Supplementary Table S3. The time evolution of ΔG_total_ (Supplementary Figure S2) shows stable envelopes during the production window, supporting the use of block-averaged statistics.

### 2.8. PCA and FEL Analysis

**Figure 9:**
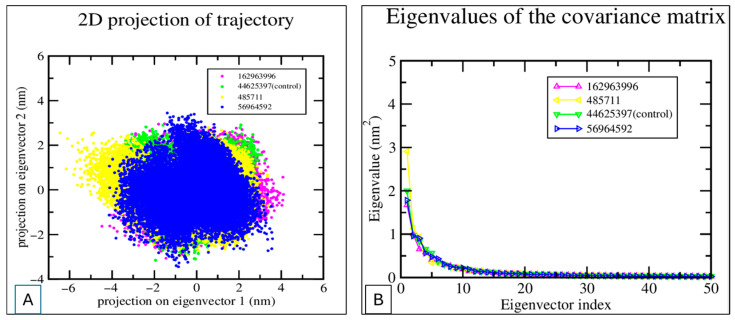
PCA of MD Trajectories. (**A**) 2D projection of trajectory snapshots onto the first two principal components (eigenvectors 1 and 2) for four systems. The distribution illustrates conformational sampling and structural diversity observed during simulations. (**B**) The eigenvalues of the covariance matrix for the four systems are plotted as a function of the eigenvector index.

**Figure 10:**
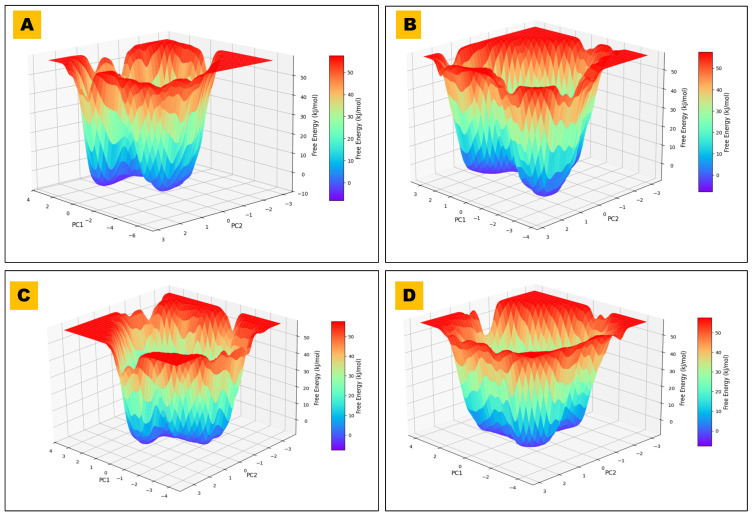
FEL of BACE1–ligand complexes. FELs are projected onto PC1–PC2 from 200 ns MD for the control and three hits. (**A**) FEL plot for BCHE1 in complex with 485711, illustrating the conformational stability and predominant energy basins observed during the simulation. (**B**) FEL plot for BCHE1 in complex with 44625397 (control compound), showing a broader and shallower energy landscape, suggesting increased conformational flexibility. (**C**) FEL plot for BCHE1 in complex with 56964592, highlighting a deep dominant energy minimum and restricted structural transitions reflecting a highly stable binding interaction. (**D**) FEL plot for BCHE1 in complex with 162963996, depicting a moderately deep central energy basin and intermediate conformational sampling characteristics.

The results of the PCA revealed significant conformational changes in BACE1-ligand complexes, with trajectories clustered along the PC1 and PC2 axes Figure 9. These key elements captured collective, slow motions relevant to protein flexibility and ligand binding. The constructed FEL plots were observed to be a well-defined energy minimum for every complex, which was also confirmed by the Free-Energy Landscape (FEL) of BACE1-Ligand Complexes, as represented in Figure 10.

It has been reported that ligands that produce deeper minima show better stability of ligand-receptor and could be associated with potent inhibitory activities. Comparative FEL analysis, as observed with the complexes of this study, revealed dissimilarities in the energy-stable conformation, supporting the selected ligand as a choice for alternative rational drug design [44].

Ligands 56964592 and 162963996 exhibited a well-defined, distinct energy minimum in the FEL plot, indicating that these molecules could attain a highly stable conformational state of BACE1 in the interacting state (complex). However, the control ligand (44625397) and other ligands 485711 and 162963996 were observed to exhibit broader distributions in PCA space and less pronounced energy wells in the FEL than Ligand 56964592, suggesting greater conformational heterogeneity in the binding state. Thus, it is concluded that the metabolites of Catharanthus, 56964592 and 162963996, showed superior binding energy and a stable, defined conformational landscape to BACE1, indicating that these could be the best candidates for further development of alternative neurotherapeutics for the management of neurological problems.

### 2.9. ADMET Profiling

**Figure 11:**
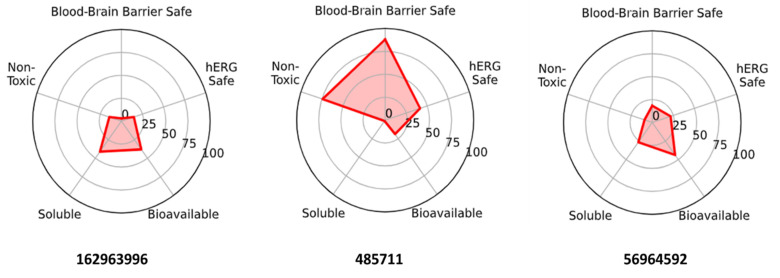
ADMET radar for the three intersection hits. Higher values indicate more favorable profiles. 162963996 and 56964592 exhibit strong BBB/bioavailability with hERG (±Ames) liabilities; 485711 shows lower hERG/Ames risk but lacks BBB permeability and solubility.

Headline CNS attributes and liabilities. 162963996 and 56964592 (catharoseumine) fall within a CNS-compatible physicochemical window (MW ≤ ~370, logP ≈ 2.4–2.5, tPSA ≈ 40–60 Å^2^) and are predicted to be BBB-permeable (≈97–100%) with moderate–good oral bioavailability. Their principal risks are ion-channel cardiotoxicity (predicted hERG liability for both) and, for 56964592, an additional AMES mutagenicity flag. In contrast, 485711 presents a low BBB probability (~19%), very poor aqueous solubility (≈1st percentile), and high lipophilicity (logP ~7.1), despite favorable hERG/AMES predictions, making it less suitable for CNS exposure without enabling strategies (e.g., prodrug/nanoformulation). Integrating virtual screening/MDS/MM-PBSA with ADMET indicates 162963996 as the primary CNS lead, 56964592 as a backup chemotype requiring de-risking for mutagenicity and hERG, and 485711 as a non-CNS-favored compound unless reformulated. These trends are summarized in Figure 11.

## 3. Materials and Methods

**Figure 12:**
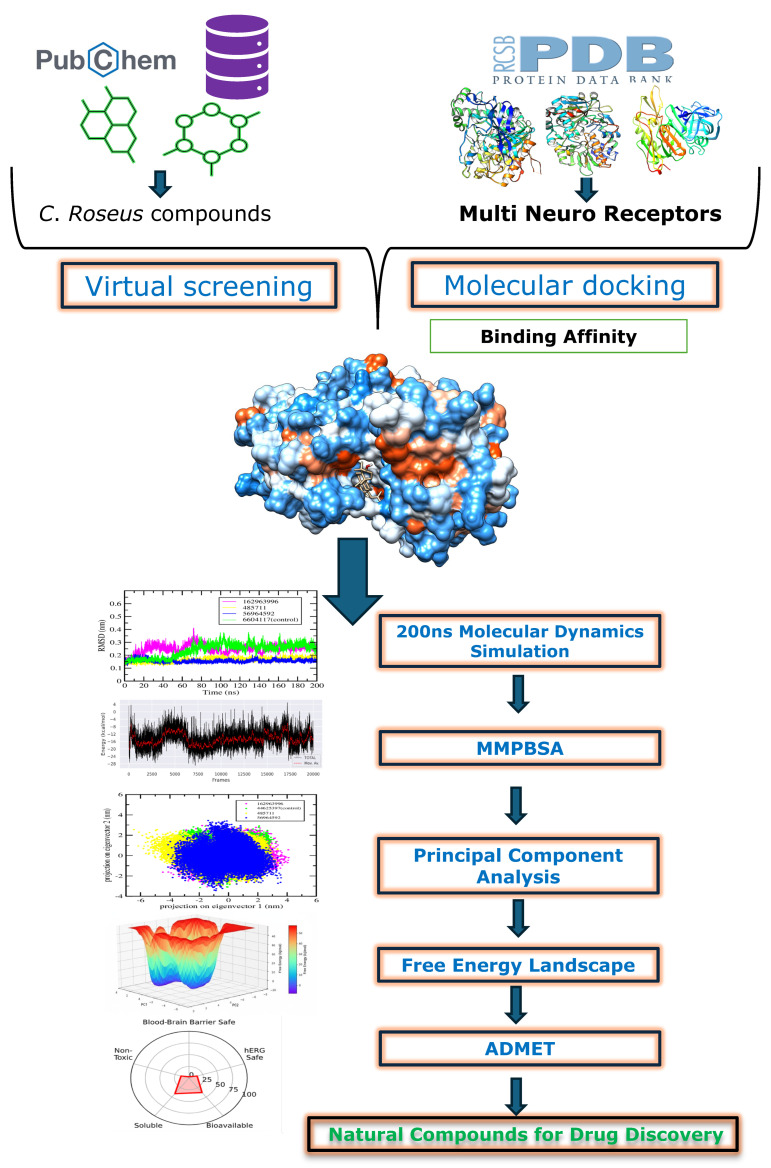
Workflow schematic of the in silico discovery pipeline.

We developed a multi-target, structure-based discovery workflow to identify neuroprotective phytochemicals from *C. roseus* that could potentially reduce disability caused by neurodegeneration. The pipeline included: (i) selecting targets and preparing receptors for five CNS proteins—AChE, BACE1, MAO-B, NMDAR, and dopamine D1; (ii) curating and preparing a library of 318 metabolites from *C. roseus*; (iii) conducting structure-based virtual screening against each target using fixed grid centers and target-specific positive controls; (iv) performing cross-target intersection analysis to find compounds consistently ranking in the top 10 for all five receptors; (v) running 200 ns all-atom MD simulations to validate docking complexes for each target; (vi) executing BACE1-focused post-docking analyses, including essential dynamics, FEL mapping, and single-trajectory MM-PB/GBSA binding-free-energy estimation; and (vii) conducting in silico ADMET profiling of the three intersection hits to assess drug-likeness, pharmacokinetics, and toxicity risks. The workflow of this study is depicted in Figure 12. Docking scores served as comparative, not exact, indicators; differences ≤ 1.0 kcal·mol^−1^ were considered ties and resolved through MD interaction persistence (top 2–3 high-occupancy contacts), MM-PB/GBSA (mean ± SEM), and ligand efficiency (LE = −ΔG_bind_/N_heavy_).

During the preparation of this manuscript, the authors used QuillBot (Version 2.0.1) for language enhancement and flow improvement. The authors have reviewed and edited the output and take full responsibility for the content of this publication.

### 3.1. Receptor Retrieval, Preparation, and Binding-Site Definition

Crystal structures were obtained from the Protein Data Bank (PDB) based on resolution, ligandability, and the preservation of active-site architecture for five CNS targets: MAO-B (2V5z) [32], AChE (4EY7), BACE1 (4D8C) [45], NMDAR (5EWJ) [46], and dopamine D1 receptor (7JOZ) [37]. Receptors were prepared using AutoDock Tools (ADT) 1.5.6 by removing non-essential crystallographic waters while retaining conserved or structural waters as needed, adding polar hydrogens, assigning Kollman charges, and accounting for alternate conformations. Binding sites were delineated based on the coordinates of the co-crystallized ligands, and the site boundaries and contacts were visually confirmed using Discovery Studio Visualizer (DSV) version v21.1.0.20298. The binding-site grid centers (*x*, *y*, *z*; in Å) used for all docking runs were: AChE: (−14.108464, −43.832714, 27.669929); MAO-B: (51.886091, 156.452818, 28.559136); BACE1: (30.577200, 6.238225, 14.522375); NMDAR: (23.531216, −5.269588, −32.403902); and Dopamine D1: (−11.853296, −82.725852, −44.039481).

All subsequent docking inputs, including receptor PDBQT files and grid definitions, were generated in ADT with consistent settings across targets to ensure methodological comparability.

### 3.2. Ligand Library Curation and Preparation

*C. roseus* metabolites (n = 318) were collated from PubChem and curated to ensure chemical consistency [47]. Records were standardized (using canonical SMILES), duplicates merged by InChIKey, and stereochemistry was retained when specified. The metabolites were downloaded into SDF format and then processed using PyRx version 0.8 [48]. Energy minimization was performed using the Universal Force Field (UFF), and the resulting minimized structures were subsequently converted to PDBQT format for further molecular docking studies [48].

### 3.3. Virtual Screening

High-throughput docking was performed in PyRx version 0.8 using AutoDock Vina version 1.1.2 as the screening engine. Binding sites were defined from the co-crystallized ligands for each target. Site centroids and grid dimensions were obtained using DSV (“Define Binding Site”) and then transferred to PyRx for batch execution. The curated *C. roseus* library (n = 318), together with target-specific positive controls, was docked to each receptor under identical settings to ensure comparability. Vina binding affinities (kcal/mol) were recorded from the top-ranked pose per ligand. Post-docking analyses were conducted in DSV to examine hydrogen bonds, hydrophobic contacts, and any other interactions with active-site residues, and to generate standardized 3D depictions of the complexes.

### 3.4. Molecular Dynamics Simulation

Docked complexes of the three intersection hits and the positive controls with two targets, AChE and BACE1, were simulated for 200 ns in GROMACS 2022 [49]. The CHARMM27 force field was used for the protein, and ligand topologies/parameters were generated with SwissParam to ensure compatibility [50]. Each complex was solvated with TIP3P water in a cubic box, with ≥1.0 nm padding between any protein atom and the box edge. Systems were neutralized with counterions (Na^+^/Cl^−^) and brought to 0.15 M NaCl to approximate physiological ionic strength.

Energy minimization was performed using steepest descents (up to 50,000 steps or until Fmax < 10.0 kJ·mol^−1^·nm^−1^). Equilibration occurred in two phases with positional restraints on solute heavy atoms: (i) NVT, 100 ps at 300 K, using the velocity-rescaling thermostat (τ = 0.1 ps), followed by (ii) NPT, 100 ps at 1 bar with the Parrinello–Rahman barostat (τ = 2.0 ps; compressibility 4.5 × 10^−5^ bar^−1^) to stabilize density.

Production trajectories were performed for 200 ns with a 2-fs time step under periodic boundary conditions. LINCS constrained all bonds involving hydrogens. Long-range electrostatics were managed using the Particle-Mesh Ewald (PME) method with rcoulomb = 1.0 nm; van der Waals interactions used a 1.0 nm cutoff. Coordinates and velocities were saved every 10 ps. Trajectory analyses were conducted with GROMACS tools: gmx rms (Cα-RMSD), gmx rmsf (per-residue RMSF), gmx gyrate (radius of gyration), and gmx hbond (hydrogen-bond counts/occupancies). Hydrogen bonds were defined by a donor–acceptor distance ≤ 0.35 nm (3.5 Å) and an angle ≥ 135°. Plots were created in XMGRACE version 5.1. 19 [51] and representative complex interactions were examined in DSV.

### 3.5. MMPBSA Calculation

Binding free energy (ΔG_bind)_ was calculated using the MM-PBSA method via the gmx_MMPBSA tool [52] and the MMPBSA.py script [53]. From the final 100 ns of each 200 ns production run, 2000 frames were extracted for analysis. Nonpolar solvation was evaluated via SASA-based terms. Reported components included ΔE_vdW_, ΔE_elec_, ΔG_gas_ (=ΔE_vdW_ + ΔE_elec_), ΔG_solv_, and ΔG_total_ (=ΔG_gas_ + ΔG_solv_); standard errors were obtained by block averaging across trajectory segments. Unless otherwise stated, entropic contributions (TΔS) were not included in ΔG_total_ because normal-mode/quasi-harmonic estimates are noisy and system-size dependent; consequently, values are best interpreted as relative free-energy trends across ligands rather than absolute affinities. Per-residue energy decomposition was performed, where indicated, to map key stabilizing contacts within the active site.

The ligand efficiency was also computed. Ligand Efficiency (LE) is calculated as the binding free energy normalized by the number of heavy (non-hydrogen) atoms:

LE = (−ΔG)/N

where ΔG is the Gibbs free energy of binding (kcal/mol) and N is the number of heavy atoms in the ligand.

### 3.6. Essential Dynamics Principal Component Analysis (PCA) and Free-Energy Landscape (FEL) Analysis

Trajectories were de-imaged, centered, and least-squares fitted to backbone heavy atoms. Principal component analysis (PCA) was performed on the covariance matrix of backbone positional fluctuations in GROMACS (gmx covar), yielding eigenvectors and eigenvalues that describe collective modes. Projections onto PC1 and PC2 (using gmx anaeig) quantified the dominant motions; the cumulative variance captured by the leading modes was recorded. The FEL on the (PC1, PC2) plane was computed at 300 K. FELs and PC scatter plots were visualized in Python version 3.9 (NumPy/matplotlib) using a perceptually ordered colormap in which cool colors indicate low free energy and warm colors high free energy. Together, PCA and FEL provide a concise view of ligand-dependent conformational stability and preferred substates of BACE1 complexes.

### 3.7. Solvent-Structure Analyses

#### 3.7.1. SASA

Solvent-accessible surface area was computed with the Shrake–Rupley algorithm (probe radius 0.14 nm), reporting protein + ligand SASA (nm^2^) using the production window (100–200 ns; frame stride matching MD analyses). Values are block-averaged over five 20 ns blocks; uncertainties are SEM.

#### 3.7.2. RDF

Radial distribution functions, *g*(*r*), were calculated with bin width 0.005 nm up to 1.2 nm for (i) water O around ligand heavy-atom centroids and (ii) ligand centroid relative to a pocket reference shell (residues defining the binding site). Peaks/decays were interpreted as structured vs. bulk-like hydration and ligand–pocket packing.

#### 3.7.3. Water Bridges

Water-mediated contacts were counted per frame when a protein–water and water–ligand hydrogen bond co-existed with geometric cutoffs (heavy-atom distance ≤ 0.35 nm; D–H···A angle ≥ 135°). We report time-fraction occupancy (%) over 100–200 ns (block-averaged; SEM).

### 3.8. In Silico ADMET Profiling

In silico ADMET profiling. The three intersection hits were evaluated with the ADMET-AI web platform [54] using PubChem SMILES as input. We recorded core CNS-relevant endpoints: blood–brain barrier (BBB) permeability (Martins), hERG I_Kr inhibition, oral bioavailability (Ma), aqueous solubility (AqSolDB percentile), and mutagenicity (AMES), alongside disposition descriptors. For visualization, probabilities were mapped to a range of 0–100, and risk endpoints were inverted to represent “safety”.

## 4. Conclusions

This integrative in silico study identifies three *C. roseus* metabolites that consistently rank among the top binders across five druggable CNS targets, namely, MAO-B, AChE, BACE1, NMDAR, and D1, supporting a multi-target mechanism relevant to disability-linked neurodegeneration. All-atom MD on AChE and BACE1 confirmed pose stability and persistent active-site contacts. On BACE1, essential dynamics/FEL analyses revealed well-defined low-energy basins, and single-trajectory MM-PBSA over the final 100 ns supported relative free-energy trends comparable to the reference inhibitor; among the hits, CID 162963996 showed the most favorable ΔG, whereas catharoseumine (CID 56964592) remained the most consistent cross-target performer (with CID 485711 showing target-selective strength). Taken together, and acknowledging the comparative nature of docking and endpoint MM-PBSA, these results nominate the three intersection hits as tractable leads for enzyme-level validation (BACE1, AChE), cell-based neuroprotection assays, and structure-guided optimization toward multi-target neurotherapeutics. We acknowledge that the lack of experimental validation is a significant limitation. Moving forward, we will conduct enzyme inhibition assays on AChE and BACE1, assess neuroprotective activity in cell-based models relevant to excitotoxicity and amyloid stress, and pursue structure-guided optimization based on pose stability and ligand-efficiency trends.

## Figures and Tables

**Table 1:** Docking metrics and key interactions of selected compounds for BACE1.

Compound	Binding Energy		Inhibition Constant	H-Bonds	H-Bond Length	Van der Waals Interactions	Pi-Pi and Other Interactions
	PyRx	AutoDock					
44625397 (control)	−8.2	−7.93	1.55 µM	A:THR72:HN–UNL1:F1	2.09712	ASN222, ARG224, SER35, ILE118, SER36, ILE126, FLN73, THR220, PHE108, LYS107, TRP115, GLY13, GLN12	Halogen TYR71 Pi-Anion ASP217 Pi-Sigma ILE110 Alkyl/Pi-Alkyl LEU30, VAL69
				A:TYR187:HH–UNL1:F2	2.57817		
				UNL1:H37–A:ASP32:OD2	2.09525		
				UNL1:H38–A:GLY34:O	2.31079		
				UNL1:H20–A:GLY219:O	2.60383		
				UNL1:C18–A:THR221:OG1	3.17536		
485711 (Epibetulinic Acid)	−9.50	−8.82	344.79 nM	UNL1:H48–A:THR72:OG1	1.98785	PRO70, GLY34, TYR71, SER35, ASP32, GLY219, LEU30, TRP115, GLN12, GLN73, THR220, ASP217, ARG224, THR318	Alkyl ILE110, ILE215, VAL321
				UNL1:H44–A:GLY11:O	2.90438		
56964592 (Catharoseumine)	−9.64	−9.17	190.18 nM	A:GLN73:HN–UNL1:O2	2.28703	ASN222, THR221, ILE110, LYS107, GLY74, PHE108, TRP115, ILE118, ASP32, SER35, THR72, GLY34, ASP217, GLY219	Pi-Pi Stacked TYR71, Alkyl/Pi-Alkyl=LEU30, TYR71
				A:THR220:CA–UNL1:O4	3.40588		
162963996	−9.20	−8.79	359.16 nM	A:THR220:HG1–UNL1:O1	3.008	ASN222, THR72, ASP217, GLY34, SER35, ILE118, LEU30, PHE108, GLY74, LYS107	Pi-Lone pair GLN73 Pi-Alkyl TYR71
				A:THR221:HN–UNL1:O1	2.25382		
				UNL1:C7–A:ASP32:OD2	2.80331		
				UNL1:C13–A:GLY219:O	3.36758		
				UNL1:C13–A:THR221:OG1	3.24096		

**Table 2:** Binding free energy components for BACE1 and ligand complexes calculated using gmx_MMPBSA with SEM and SD. Energies are in kcal/mol.

Complex	ΔVDWAALS	ΔEEL	ΔG_gas_	ΔG_solv_	ΔG_total_	SEM	SD
Control	−20.42	−242.74	−263.16	242.31	−20.85	±0.07	±9.59
485711	−24.16	−9.22	−33.38	21.17	−12.2	±0.03	±4.91
56964592	−36.25	−143.62	−179.88	165.23	−14.64	±0.03	±4.29
162963996	−28.04	−214.79	−242.84	226.75	−16.09	±0.05	±6.85

## Data Availability

The original contributions presented in this study are included in the article and Supplementary Materials. Further inquiries can be directed to the corresponding author.

## Supplementary Materials

The following supporting information can be downloaded at: https://www.mdpi.com/article/10.3390/ph18111734/s1, Figure S1: Water-bridge dynamics. Protein–water, water-mediated (protein–water–ligand), and ligand–water timelines for the four BACE1 systems. Figure S2: ΔG_total_ time-evolution. Trajectory-wise ΔG_total_ profiles (black) with moving averages (red) for control and three hits. Figure S3: Interaction occupancy. Time-fraction contact/selection occupancy for control and hits for BACE 1. Figure S4: SASA over time. Complex SASA traces (nm^2^) across 200 ns for control and hits for BACE 1. Figure S5: RDFs. g(r) of ligand centroids vs. pocket reference over 200 ns for BACE 1. Figure S6. Dopamine D1 Receptor MD panels (200 ns). RMSD, Rg, RMSF, H-bond counts (control + hits). Figure S7: MAO-B MD panels (200 ns). RMSD, Rg, RMSF, H-bond counts (control + hits). Figure S8: MDS analyses of the protein in complex with selected compounds (162963996, 485711, 56964592) and control (6604117) over 200 ns; Table S1: IUPAC names and 2D structures of the three intersection hits; Table S2: Binding free energy components for whole complexes (Poisson-Boltzmann Model) with SEM and SD; Table S3: Summary of binding free energies (ΔG_bind_), number of heavy atoms (N_heavy_), and ligand efficiency (LE) for selected compounds.
