# Supplementary material for: Catharanthus roseus Phytochemicals as Multi-Target Modulators of Disability-Linked Neurodegeneration: Bio-Computational Insights
Source: Pharmaceuticals (Basel). 2025 Nov 14;18(11):1734. doi: 10.3390/ph18111734 (PMC12655304; doi:10.3390/ph18111734)
Supplement: Supplementary file 1 [file pharmaceuticals-18-01734-s001.zip › pharmaceuticals-3927370-supplementary.pdf]

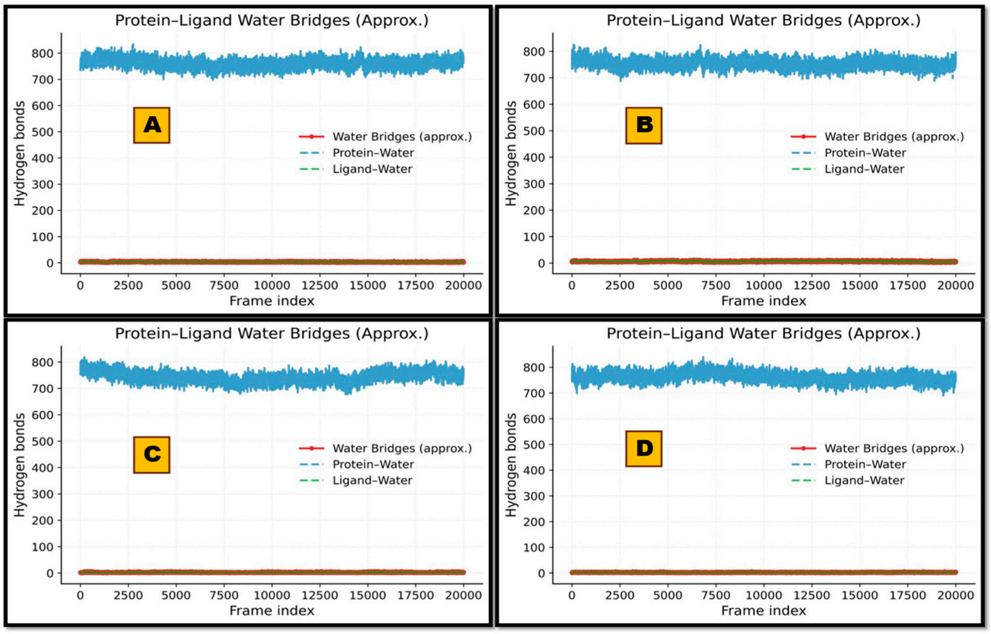

Figure S1: Water-bridge dynamics. Protein–water, water-mediated (protein–water–ligand), and ligand–water timelines for the four BACE1 systems.

(**A**) A-485711; (**B**) 44625397 (control); (**C**) 56964592; (**D**) 162963996.
Red line: approximate water bridges (protein–water–ligand); blue dashed line: protein–water; green dashed line: ligand–water.

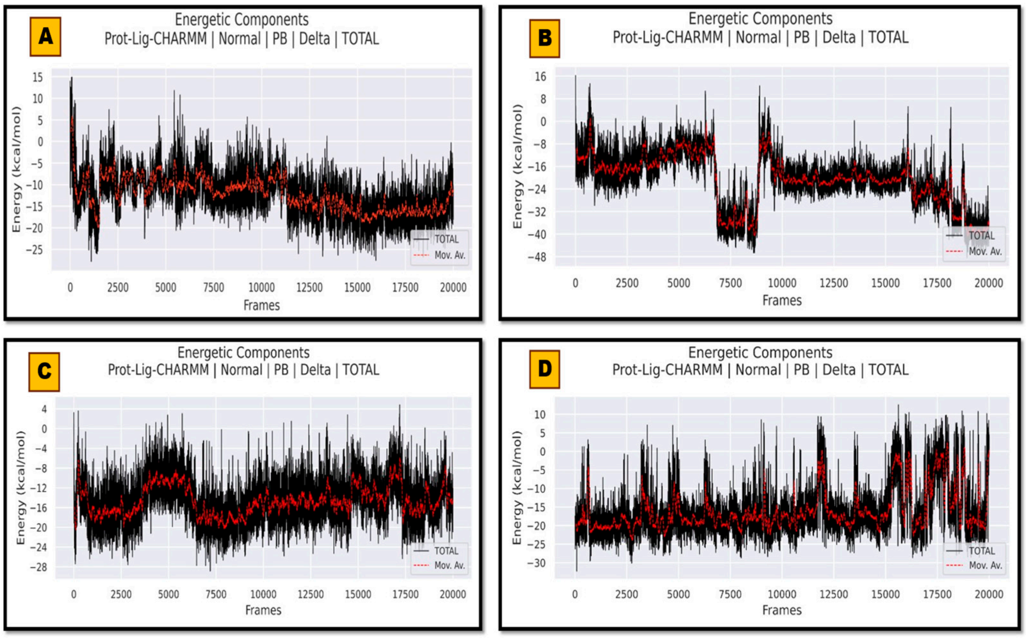

Figure S2. ΔG_{total} time-evolution. Trajectory-wise ΔG_{total} profiles (black) with moving averages (red) for control and three hits.

(**A**) A-485711; (**B**) 44625397 (control); (**C**) 56964592; (**D**) 162963996, calculated over 20,000 MD simulation frames. Consistent negative energies and reduced fluctuations characterize the strong, stable binding observed with 56964592 and 162963996.

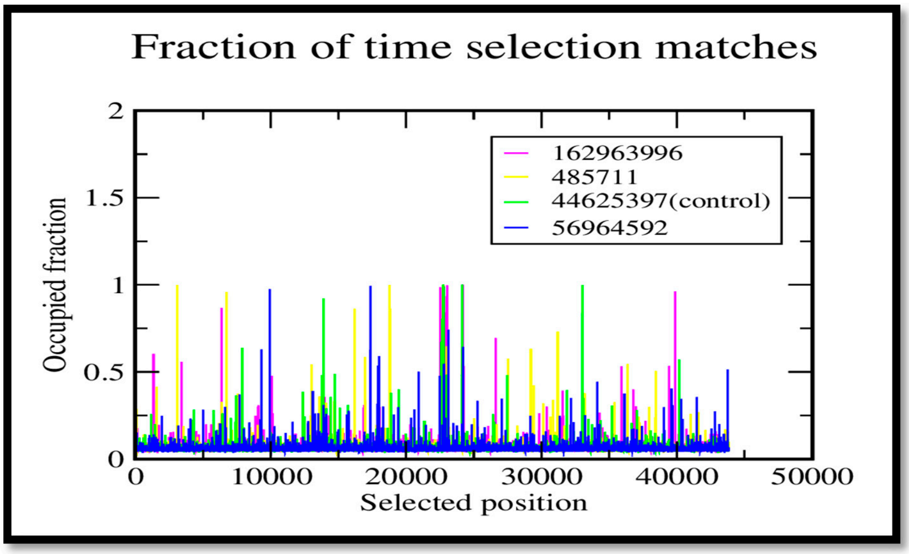

Figure S3. Interaction occupancy. Time-fraction contact/selection occupancy for control and hits for BACE 1.

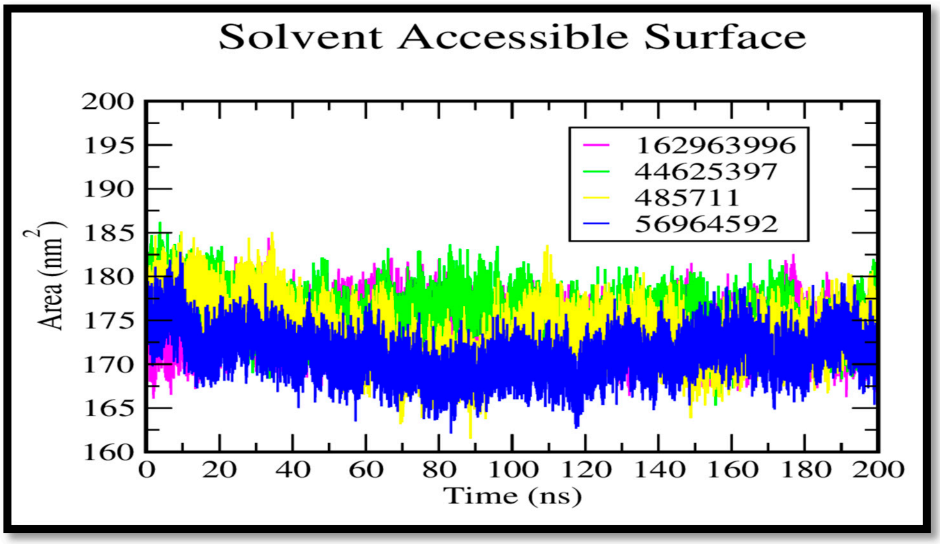

Figure S4. SASA over time. Complex SASA traces (nm²) across 200 ns for control and hits for BACE 1.

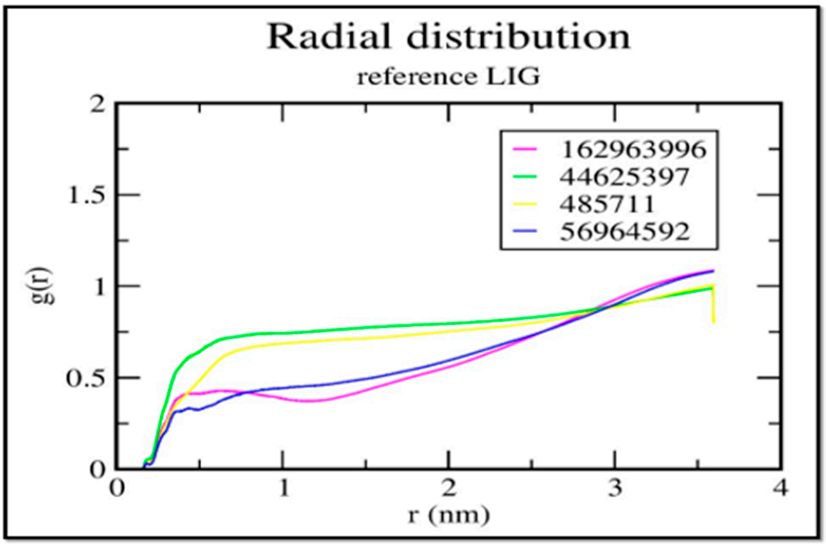

Figure S5: RDFs. g(r) of ligand centroids vs. pocket reference over 200 ns for BACE 1.

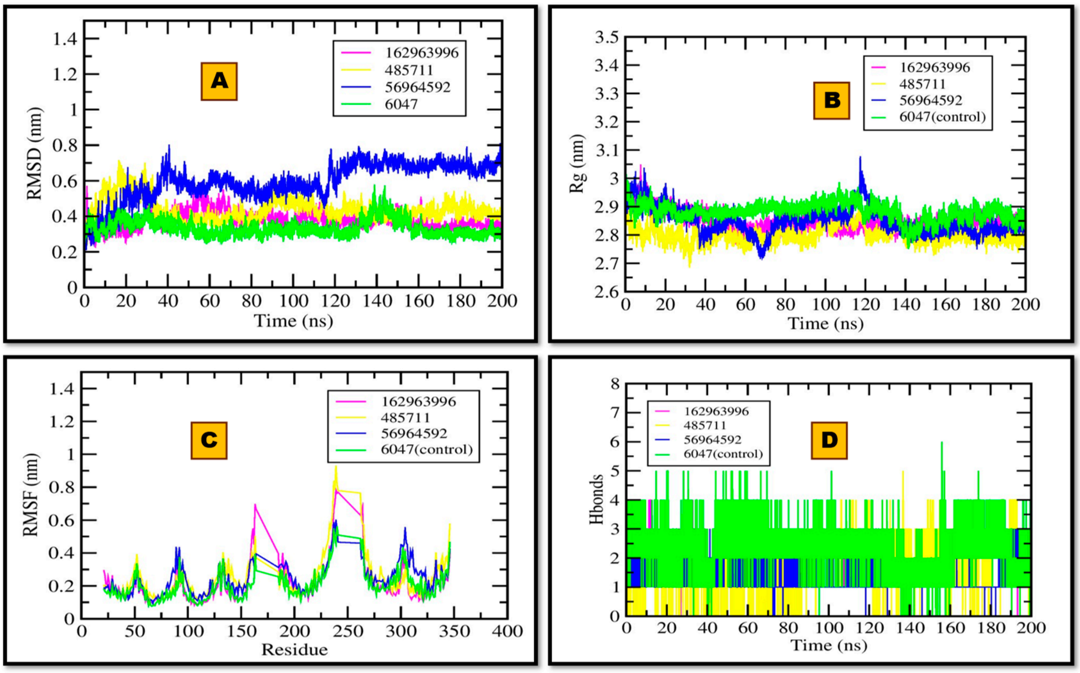

Figure S6. Dopamine D1 Receptor MD panels (200 ns). RMSD, Rg, RMSF, H-bond counts (control+hits).

(**A**) RMSD values, (**B**) radius of gyration, (**C**) RMSF, and (**D**) hydrogen bonds during simulations.

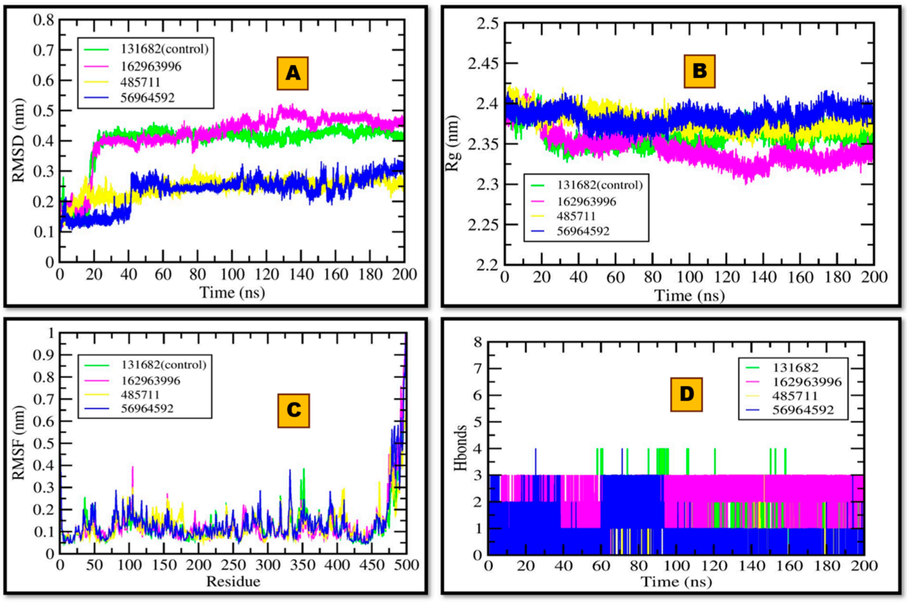

Figure S7. MAO-B MD panels (200 ns). RMSD, Rg, RMSF, H-bond counts (control + hits).

(**A**) RMSD values, (**B**) radius of gyration, (**C**) RMSF, and (**D**) hydrogen bonds during simulations.

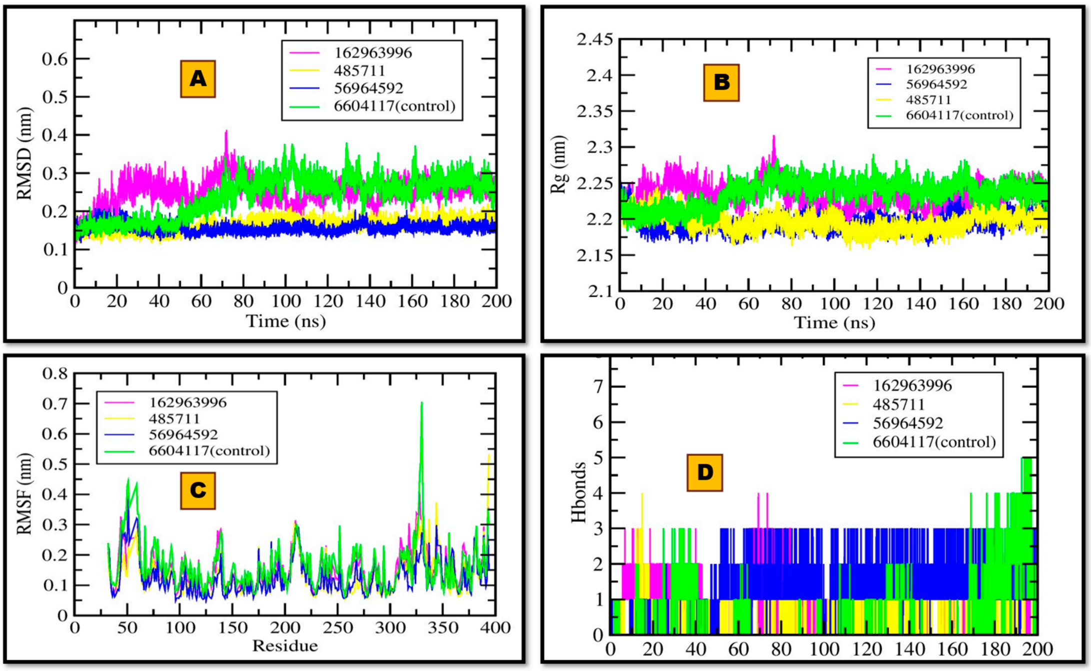

Figure S8. MDS analyses of the protein in complex with selected compounds (162963996, 485711, 56964592) and control (6604117) over 200 ns.

(**A**) RMSD; (**B**) radius of gyration (Rg); (**C**) residue-wise RMSF; (**D**) protein-ligand hydrogen bonds.

Table S1. IUPAC names and 2D structures of the three intersection hits

Compounds	IUPAC Name	2D structure
485711	(1*R*,3*aS*,5*aR*,5*bR*,7*aR*,9*R*,11*aR*,11*bR*,13*aR*,13*bR*)-9-hydroxy-5*a*,5*b*,8,8,11*a*-pentamethyl-1-prop-1-en-2-yl-1,2,3,4,5,6,7,7*a*,9,10,11,11*b*,12,13,13*a*,13*b*-hexadecahydrocyclopenta[a]chrysene-3*a*-carboxylic acid	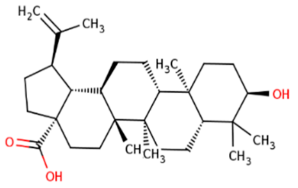
56964592	methyl (1*R*,12*S*,16*S*,19*S*)-19-methyl-17,18-dioxa-5,15-diazahexacyclo[13.4.3.$0^{1,16}$.$0^{4,12}$.$0^{6,11}$.$0^{12,16}$]docosa-3,6,8,10,20-pentaene-3-carboxylate	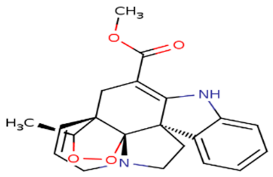
162963996	Methyl 20-methyl-8,16-diazahexacyclo[10.6.1.19,11.01,9.02,7.016,19]icosa-2,4,6,13-tetraene-10-carboxylate	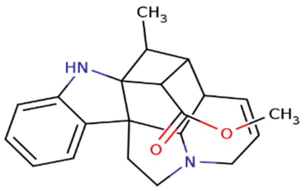

Table S2. Binding free energy components for whole complexes (Poisson-Boltzmann Model) with SEM and SD

Complex	ΔVDWAALS	ΔEEL	ΔG_{gas}	ΔG_{solv}	ΔG_{total}	SEM	SD
**Control**	-2608.60	-23321.71	-2075.60	-4357.14	-6432.75	±0.44	±62.37
**485711**	-2609.71	-23010.55	-1949.71	-4455.42	-6405.13	±0.44	±62.75
**56964592**	-2646.45	-23023.44	-2106.41	-4396.08	-6502.48	±0.47	±66.58
**162963996**	-2620.01	-23101.67	-2070.14	-4367.79	-6437.94	±0.42	±59.47

Table S3: Summary of binding free energies (ΔG_{bind}), number of heavy atoms (N_{heavy}), and ligand efficiency (LE) for selected compounds

complex	MMPBSA energies (Kcal/mol) (ΔG_{bind})	Number of Heavy atoms (N_{heavy})	Ligand efficiency (LE)
Control	-20.85	44	-0.47
485711	-12.21	35	-0.35
56964592	-14.64	28	-0.52
162963996	-16.09	26	-0.61

Ligand efficiency can be calculated as $LE = -\Delta G_{bind}/N_{heavy}$
